# Strategies for evaluating self-efficacy and observed success in the practice of yoga postures for therapeutic indications: methods from a yoga intervention for urinary incontinence among middle-aged and older women

**DOI:** 10.1186/s12906-020-02934-3

**Published:** 2020-05-14

**Authors:** Francesca M. Nicosia, Nadra E. Lisha, Margaret A. Chesney, Leslee L. Subak, Traci M. Plaut, Alison Huang

**Affiliations:** 1 Division of Geriatrics and Institute for Health & Aging, University of California, San Francisco, USA; 2 San Francisco Veterans Affairs Health Care System, 4150 Clement Street, 151-R, San Francisco, CA 94121 USA; 3 Center for Tobacco Control Research and Education and Division of General Internal Medicine, University of California San Francisco, 530 Parnassus, Ste 366, San Francisco, CA 94143-1390 USA; 4 Department of Medicine and Osher Center for Integrative Medicine, University of California San Francisco, 1545 Divisadero, San Francisco, CA 94118 USA; 5 Department of Obstetrics and Gynecology, Stanford University School of Medicine, 300 Pasteur Drive, HG332, Office #G-303A, Stanford, California, 94305-5317 USA; 6 Division of General Internal Medicine, University of California San Francisco, Street Suite 201, Sutter, 2320 USA; 7 Division of General Internal Medicine, University of California San Francisco, 1545 Divisadero Street, San Francisco, CA 94118 USA

**Keywords:** Yoga, Self-efficacy, Urinary incontinence, Integrative medicine, Clinical trial, Research methodology, Patient adherence

## Abstract

**Background:**

Most clinical investigations involving yoga lack adequate description of the specific yoga elements, including physical postures. Few studies have measured self-efficacy regarding the performance of yoga postures or assessed observed success in performing postures.

**Methods:**

We developed and piloted several tools to evaluate self-efficacy and observed success in practicing yoga in the context of a randomized feasibility trial of an Iyengar-based yoga intervention for urinary incontinence in ambulatory women ≥50 years. At the end of the 12-week yoga intervention involving twice weekly group yoga classes and once weekly home practice, participants rated their self-efficacy in performing each of the included 15 yoga postures on a 5-point Likert scale. During the 12th week, an expert yoga consultant observed participants and rated their competency in performing postures on a 5-point scale. Participants completed a questionnaire about self-efficacy in adhering to home yoga practice. We examined the distribution of and correlations between scores on the above measures.

**Results:**

Among 27 participants (mean age 65 years), the range of means for self-efficacy ratings for individual postures was 3.6 to 4.5. The range of means for observed competency ratings for individual postures was 3.3 to 5.0. Mean self-efficacy rating for confidence in adhering to the assigned once-weekly home yoga practice was 2.8 (range 1 to 5). Posture self-efficacy was inversely correlated with participant age (*p* = 0.01) and positively correlated with self-reported physical function (*p* = 0.03) and mobility (p = 0.01). No significant correlations were found between posture self-efficacy scale scores and expert-observed yoga competency ratings or practice adherence self-efficacy scores.

**Conclusions:**

These measures hold promise for advancing yoga research and practice by describing methods to: 1) measure self-efficacy in performing specific yoga postures; 2) use an expert observer to assess participants’ competence in performing yoga postures; and 3) measure self-efficacy in adhering to home practice. These proposed measures can be used to describe specific components of yoga interventions, to assess whether study participants are able to learn to practice physical aspects of yoga and/or maintain this practice over time, as well as to investigate relationships between self-efficacy and competency in performing yoga postures to achieve specific health outcomes.

**Trial registration:**

ClinicalTrials.gov, NCT02342678, January 21, 2015.

## Background

Yoga interventions to improve health and wellbeing are being developed and implemented with increasing frequency. Over the past two decades, the number of clinical investigations and randomized controlled trials involving yoga to treat or prevent specific health conditions has risen dramatically. However, reports of many of these interventions are limited by inadequate description of the specific type or elements of yoga used, [1, 2] including practice of physical yoga asanas (postures) [3, 4]. In a recent systematic review, fewer than 50% of published clinical trials of yoga interventions described the specific yoga postures used in the study protocol [3]. Furthermore, few studies have attempted to assess the success of study-specific yoga instruction by examining indicators such as participants’ perceived or observed success [3, 5–8] in practicing specific yoga postures for therapeutic purposes.

Self-efficacy is defined as a belief about one’s ability to perform a specific behavior or “the conviction that one can successfully execute the behavior required to produce (certain outcomes)” [9]. Thus, beliefs about one’s ability to perform specific yoga postures may influence outcomes of yoga interventions designed to improve health. Recently developed and tested measures of self-efficacy for yoga research have focused on mental or psychological aspects of yoga, including the Yoga Self-Efficacy Scale developed to assess self-efficacy related to overall ability to “focus body, breath and mind” [6, 10, 11]. Rarely have studies measured self-efficacy regarding the physical performance of specific yoga postures to improve health conditions. Few studies have taken the further step of assessing whether self-efficacy corresponds to participants’ observed success in performing postures [12, 13].

To better understand the mechanisms responsible for the observed health effects of yoga, there is a need for external assessment of participants’ competence in performing physical yoga postures in accordance with research protocols. To date, studies have not described methods for critical observation of study participants’ competence in performing yoga postures [14].

While some forms of yoga place relatively little emphasis on the precision of execution of yoga postures, Iyengar yoga, one of the most common style of yoga studied in randomized trials, [3] is known for its systematic emphasis on precise anatomic alignment during the practice of postures [15, 16]. Iyengar yoga is therefore particularly suited to developing measures to assess self-efficacy of physical performance of postures for therapeutic purposes. Thus we developed several new tools to evaluate self-efficacy and observed competency in performing yoga postures and evaluated them in a pilot randomized trial of a therapeutic Iyengar-based yoga intervention for urinary incontinence in middle-aged and older women [17]. This study does not present clinical outcomes but instead presents unpublished methodology for measuring self-efficacy and observed success in yoga-based interventions. Development of such process measures will help advance research on therapeutic yoga interventions by 1) describing specific components of yoga interventions and 2) providing researchers with data on participants’ self-efficacy in performing yoga postures and home practice adherence or their objective ability to successfully perform the postures, and whether or not these are related to the fidelity and effectiveness of the intervention.

## Methods

### Aims

Our goals were to develop new process measures designed to: 1) assess participants’ self-efficacy in performing specific yoga postures; 2) pilot-test a new expert consultant method for rating study participants’ competence in performing yoga postures; and 3) advance methods for measuring self-efficacy in adherence to ongoing practice of yoga.

### Setting and participants

The Lessening Incontinence through Low-Impact Activity (LILA) study was a randomized trial designed to assess the feasibility of recruiting and retaining ambulatory middle-aged and older women in a 3-month Iyengar-based yoga program for urinary incontinence, assess the tolerability and acceptability of yoga in this population, and evaluate preliminary changes in incontinence with yoga practice [18]. Participants were recruited from the San Francisco Bay area community from 2015 to 2017. Written informed consent was obtained from all participants before enrollment, study procedures were approved by University of California, San Francisco Institutional Review Board (#14–14,732), and the trial was registered in ClinicalTrials.gov.

Details of the LILA study design and eligibility criteria are reported elsewhere [17, 18]. Briefly, participants were ambulatory women at least 50 years of age who reported at least daily stress-, urgency- or mixed type urinary incontinence, did not have complex urologic or neurologic histories, and were willing to forgo standard clinical incontinence treatments during the trial period. To be eligible, women had to be able to walk two blocks on level ground and transition from a supine to a standing position without assistance. Women could not already be engaged in organized yoga classes or previously have completed yoga training directed specifically at incontinence.

### Yoga intervention

The therapeutic yoga program was designed to provide instruction and practice in selected yoga postures and techniques chosen for their potential to improve bladder control in older women as well as promote safety and feasibility in this population. The program was based in Iyengar yoga, a form of Hatha yoga that is known for its potential therapeutic applications, has been employed successfully in other studies of yoga for other indications, [19–24] and differs from other yoga styles in ways that were thought likely to maximize both efficacy, safety and accessibility. These included: 1) emphasis on precise anatomical alignment and awareness of specific bodily structures during practice of yoga postures; 2) incorporation of props to minimize risk of injury, accommodate those with decreased strength or flexibility, and increase comfort and duration of postures; and 3) emphasis on mindful awareness rather than rapid cycling through postures.

The program focused on a core set of 15 yoga postures commonly used in Hatha yoga practice, including active postures to engage the pelvic floor and passive, supported postures to promote relaxation (Additional Table 1). During each intervention wave (4 total), participants engaged in twice weekly 90-min group classes with an average of 6 to 8 women, led by an instructor with at least 2 years of experience teaching yoga in community settings who had undergone study-specific training with the study expert yoga consultant. The instructor followed a study-specific guide to introduce participants to yoga postures, calling attention to ways in which postures could improve pelvic floor function, and guiding women in adapting postures and using props to accommodate physical limitations as needed. Participants were also asked to practice yoga at home at least one additional hour per week. In addition, women were given a written manual that included pictures and descriptions of each posture to guide them in home practice, as well as a yoga mat, belt, and two blocks for home practice.

### Yoga posture self-efficacy assessment (Y-SEA)

At the end of the intervention program (week 12), participants were asked to complete a self-assessment instrument in which they rated their confidence in performing each of the yoga postures featured in the LILA yoga program and holding it for 30 s, using a 5-point scale (5-extremely, 4-very, 3-moderately, 2-somewhat, and 1-not at all confident). This instrument was adapted from an existing measure for assessing self-confidence in practicing Hatha yoga to improve self-esteem in menopausal women [11]. Participants were told that if they had been taught a modified form of a posture rather than the standard version (such as using props to accommodate limitations in flexibility), they should rate their confidence in performing the modified rather than standard version. The instrument included pictures, and Sanskrit and English names of each posture.

### Expert-observed yoga competency assessment

During week 12, a study expert yoga consultant visited each group class to assess participants’ competency in performing yoga postures. The expert consultant had over 3500 h of training, including over 2000 h of training in the Iyengar method, and over 20 years teaching experience. In addition, she was certified through the International Association of Yoga Therapists and was an author and teacher trainer for yoga for pelvic floor health. The consultant let the participants know that she was observing the class in order to assess the quality and success of yoga instruction at the end of the study yoga program, without emphasizing that this included specific assessment of participants’ execution of study-specific yoga poses. The consultant observed each participant attending the class while moving around the room and rated their success in performing each yoga posture on a 5-point scale (5-extremely, 4-very, 3-moderately, 2-somewhat, and 1- not at all). A variety of factors were considered to determine participants’ competency with specific poses including form and alignment, ease and quality of breathing, ability to follow instructions and hold a pose for the suggested duration, and overall posture difficulty level (e.g., standing versus supine). If the participant performed a modified version of the posture, the expert was instructed to rate the performance of the modified rather than the standard version.

### Yoga practice adherence self-efficacy (Y-PASE)

Participants were asked to rate their self-efficacy in being able to adhere to regular practice of yoga at week 12 using a questionnaire modeled after existing physical activity/exercise adherence self-efficacy scales [25]. Women indicated on a 5-point scale how confident they were that they could practice yoga when they: 1) are tired, 2) are in a bad mood, 3) have limited time, 4) are away from home, and 5) are not regularly attending yoga classes.

### Other measures

To assess change in incontinence symptoms, participants also completed several measures at baseline and 12 weeks. Frequency of incontinence was assessed using validated 3-day voiding diaries in which participants recorded all incontinence episodes over a 3-day period [26]; diary data were then abstracted by blinded analysts. Participants also completed the Patient Perception of Bladder Condition (PPBC), a single-item measure assessing the degree to which respondents consider their bladder condition to be a problem on a 6-point Likert scale, with higher scores indicating more bladder problems [27].

To assess depression and anxiety, participants completed questionnaires at baseline and 12 weeks: 1) the Hospital Anxiety and Depression Scale (HADS) – Depression Subscale, a 7-item measure of depression, including loss of interest in pleasurable activities; and 2) the Hospital Anxiety and Depression Scale (HADS) – Anxiety Subscale, a 7-item measure of cognitive anxiety associated with fear of failure [28]. Scores range from 0 to 21, with higher scores indicating greater anxiety.

Physical function and performance were measured with the: 1) PROMIS SF-8 Adult Physical Function Profile short-form, a questionnaire measure of the impact of physical function on activities of daily living, lower extremity, and central body functions, scaled from 0 to 100, with higher scores indicating greater self-reported function; and 2) Short Physical Performance Battery (SPPB) score, a series of physical performance tests to assess lower extremity functioning, scored from 0 to 12, with higher scores indicating better functioning.

### Analysis

One-way frequency tables were computed for all analysis variables and measures of central tendency were computed for continuous variables. In addition to examining participant self-efficacy ratings associated with each individual posture, we calculated a combined/cumulative yoga posture self-efficacy score based on the average of all individual posture self-efficacy scores. We also examined the proportion of participants who indicated that they were at least 1) “moderately confident” or at least 2) “very confident” in their ability to perform all postures.

The same approach was used to examine data distribution from the expert-observed yoga competency assessment. In addition to examining participants’ observed success in performing each individual posture, we calculated a combined/cumulative competency assessment score based on the average of the observed competency scores for all individual postures.

A total Y-PASE score was calculated as the average of each of the five individual questions about self-efficacy in adherence to yoga practice. To ensure that the Y-PASE was indeed a single factor, an exploratory factor analysis was performed. Factors with Eigenvalues under 1 were not retained. Internal consistency for these scales was calculated using Cronbach’s alpha [29, 30].

Lastly, we examined correlations between scores on the three main scales (Y-SEA, expert-observed competency assessment score, and Y-PASE) with selected demographic and clinical characteristics, including age, absolute change in urinary incontinence frequency over 12 weeks, participant perception of bladder control, depression symptoms, anxiety symptoms, and self-reported and observed physical function at 12 weeks.

## Results

### Participant characteristics (Table 1)

Of the 28 women assigned to the yoga group, mean age (standard deviation) was 65.0 (±8.9), and 47% were racial or ethnic minorities. Two thirds reported urgency-predominant incontinence, and 44% reported having incontinence for five or more years. Average scores on questionnaire measures for anxiety and depression symptoms at baseline were below standard thresholds for having clinically significant anxiety or depression [28]. Average scores on measures for physical function indicated that few participants had compromised physical function.

**Table 1:** Baseline demographic and clinical characteristics of participants in the yoga group

	Yoga Group (*N* = 27)
Demographic history	
Age in years	65.0 (±8.9)
Age ≥ 65 years	11 (40.7%)
Race/ethnicity	
Non-Latina White	17 (63.0%)
Non-Latina African-American	0 (0%)
Latina	1 (3.7%)
Non-Latina Asian/Asian-American	4 (14.8%)
Non-Latina Native Hawaiian or other Pacific Islander	2 (7.4%)
Unknown	3 (11.1%)
Gynecologic history	
Hysterectomy (known risk factor for UI)	5 (18.5%)
General medical history	
Heart disease	1 (3.7%)
Lung disease (asthma, bronchitis, COPD)	7 (25.9%)
Arthritis	13 (48.2%)
Health-related habits	
Current smoker	2 (16.7%)
≥ 1 Alcoholic beverage per week	14 (51.9%)
Physical exam measures	
Body mass index (kg/m^2^)	26.5 (±4.4)
Clinical incontinence type	
Urgency or urgency-predominant	18 (66.7%)
Stress or stress-predominant	9 (33.3%)
Equally mixed stress-and-urge	0 (0%)
Incontinence frequency (episodes/day)	
Total Incontinence	3.9 (±1.5)
Urgency Incontinence	2.2 (±1.8)
Stress Incontinence	1.4 (±1.6)
Incontinence duration	
Less than 1 year	2 (7.4%)
1 to 4 years	13 (48.2%)
5 years or more	12 (44.4%)
Anxiety, depression, & sleep questionnaire scores	
Hospital Anxiety and Depression Scale (HADS) – Anxiety Subscale	5.8 (±4.6)
Hospital Anxiety and Depression Scale (HADS) – Depression Subscale	3.5 (±3.2)
Physical function and performance	
PROMIS Adult Physical Function Profile short-form	13.8 (±5.6)
Short Physical Performance Battery (SPPB) score	11.5 (±1.3)

Data are presented as number (percentage) or mean (±standard deviation)

### Yoga posture self-efficacy assessment (Y-SEA) (Table 2)

The mean self-efficacy rating for individual postures (Table 2) ranged from 3.6 to 4.5. The three postures associated with the highest mean self-efficacy ratings were Savasana (corpse pose, 4.7), Tadasana (mountain pose, 4.5), and Supta padangusthasana (reclined hand to big toe pose, 4.5). The three postures associated with the lowest self-efficacy ratings were Malasana (squat/garland pose, 3.6), Salabhasana (locust pose, 3.9), and Utkatasana (chair pose, 4.0). Seventy four percent of participants reported being at least moderately confident, and 22% reported being very confident in their ability to perform all postures in the program. Cronbach alpha for this scale was 0.93.

**Table 2:** Participants’ Self-Confidence in Performing Specific Yoga Poses at Week 12, Based on the Yoga Posture Self-Efficacy Assessment Questionnaire (Y-SEA)

	Percentage of participants reporting confidence in performing each posture					
Posture	Not at all confident [1]	Slightly confident [2]	Moderately confident [3]	Very confident [4]	Extremely confident [5]	Mean self-efficacy rating^a^ (SD)
Tadasana (mountain pose)	0%	0%	7.4%	33.3%	59.3%	4.5 (0.6)
Utkatasana (chair pose)	3.7%	3.7%	14.8%	40.7%	37.0%	4.0 (1.0)
Trikonasana (triangle pose)	0%	7.4%	18.5%	29.6%	44.4%	4.1 (1.0)
Virabhadrasana 2 (warrior 2 pose)	0%	3.7%	14.8%	29.6%	51.0%	4.3 (0.9)
Parsvottanasana (intense side stretch pose)	0%	3.7%	14.8%	37.0%	44.4%	4.2 (0.8)
Malasana (garland/squat pose)	7.4%	7.4%	29.6%	25.9%	29.6%	3.6 (1.2)
Bharadvajasana (seated twist pose)	0%	3.7%	11.1%	25.9%	59.3%	4.4 (0.8)
Vajrasana	0%	3.7%	14.8%	33.3%	48.2%	4.3 (0.9)
Baddha Konasana (bound angle pose)	0%	0%	14.8%	25.9%	59.3%	4.4 (0.8)
Shalabhasana (locust pose)	0%	7.4%	29.6%	25.9%	37.0%	3.9 (1.0)
Salamba setubandhasana (supported bridge pose)	0%	0%	25.9%	25.9%	48.2%	4.2 (0.8)
Supta padangusthasana (reclined hand to big toe pose)	0%	0%	11.1%	25.9%	63.0%	4.5 (0.7)
Supta baddha konasana (reclined bound angle pose)	0%	0%	11.1%	29.6%	59.3%	4.5 (0.7)
Viparita karani variation (inverted lake pose)	3.7%	3.7%	3.7%	29.6%	59.3%	4.4 (1.0)
Savasana (corpse pose)	0%	0%	3.7%	18.5%	77.8%	4.7 (0.5)

Percentages are column percentages

^a^Calculated by taking the average of all self-efficacy ratings for each posture, based on a scale of 1 (not at all confident) to 5 (extremely confident)

### Expert-observed yoga competency ratings (Table 3)

Mean observed participant competency ratings for individual yoga postures (Table 3) ranged from 3.3 to 5.0. The three postures associated with the highest mean observed competency ratings were Savasana (corpse pose, 5.0), Supta padangusthasana (reclined hand to big toe pose, 4.6), and Tadasana (mountain pose, 4.5). The three postures associated with the lowest mean observed competency ratings were Trikonasana (triangle pose, 3.3), Virabhadrasana 2 (warrior 2, 3.8), and Salambha setubhandasana (supported bridge pose, 3.9). The expert observer rated 26% of participants at least very successful for all poses and 96% at least moderately successful for all poses. Cronbach alpha for this scale was 0.93.

**Table 3:** Participants’ observed competence in performing specific yoga postures at 12 weeks, based on expert evaluation

	Percentage of participants receiving each competency rating for each posture^a^					
Posture	Not at all successful [1]	Slightly successful [2]	Moderately successful [3]	Very successful [4]	Extremely successful [5]	Mean competency rating^b^ (SD)
Tadasana (mountain pose)	0%	0%	0%	47.8%	52.2%	4.5 (0.5)
Utkatasana (chair pose)	0%	0%	4.4%	47.8%	47.8%	4.4 (0.6)
Trikonasana (triangle pose)	0%	4.4%	69.6%	17.4%	8.7%	3.3 (0.7)
Virabhadrasana 2 (warrior 2 pose)	0%	4.4%	39.1%	30.4%	26.1%	3.8 (0.9)
Parsvottanasana (intense side stretch pose)	0%	4.4%	0%	65.2%	30.4%	4.2 (0.7)
Malasana (garland/squat pose)	4.6%	0%	9.1%	27.3%	59.1%	4.4 (1.0)
Bharadvajasana (seated twist pose)	0%	0%	21.7%	30.4%	47.8%	4.3 (0.8)
Vajrasana	0%	0%	34.8%	13.0%	52.2%	4.2 (0.9)
Baddha Konasana (bound angle pose)	0%	0%	22.7%	18.2%	59.1%	4.4 (0.8)
Shalabhasana (locust pose)	5.0%	0%	0%	30.0%	65.0%	4.5 (0.6)
Salamba setubandhasana (supported bridge pose)	5.0%	0%	0%	30.0%	65.0%	3.9 (1.0)
Supta padangusthasana (reclined hand to big toe pose)	0%	0%	4.4%	34.8%	60.9%	4.6 (0.6)
Supta baddha konasana (reclined bound angle pose)	0%	0%	0%	52.2%	47.8%	4.5 (0.5)
Viparita karani variation (inverted lake pose)	0%	0%	12.5%	31.3%	56.3%	4.4 (0.7)
Savasana (corpse pose)	0%	0%	0%	0%	100%	5.0 (0)

Percentages are column percentages

^a^4 (14.8%) women did not have expert observation at all, 13 (48%) had data on all postures, remaining women were missing between 1 and 3 postures

^b^Calculated by taking the average of all participant competency ratings for each posture, based on a scale of 1 (not at all successful) to 5 (extremely successful

### Yoga practice adherence self-efficacy (Y-PASE) (Table 4)

Factor analysis of the Y-PASE questionnaire indicated one factor (eigenvalue factor 1 = 1.78, eigenvalue factor 2 = 0.21). The mean score obtained on the Y-PASE questionnaire was 2.8 (range 1 to 5). Mean scores on each individual Y-PASE item ranged from 2.3 to 3.3. The overall standardized Cronbach alpha was 0.68, indicating moderate internal consistency reliability. Item-scale correlations for “When I am on vacation or away from home” was low (0.2), while all other items were moderate to high (0.37–0.58).

**Table 4:** Distribution of participant responses to the yoga practice adherence self-efficacy (y-pase) questions at week 12

	How confident are you that you would practice yoga in each of the following situations?				
Response frequencies	When I am tired	When I am in a bad mood	When I don’t have time	When I am on vacation or away from home	When I am not regularly attending classes
(1) Not at all confident	5 (18.5%)	2 (7.4%)	5 (18.5%)	4 (14.8%)	1 (3.7%)
(2) Slightly confident	8 (29.6%)	5 (18.5%)	10 (37.0%)	5 (18.5%)	3 (11.1%)
(3) Moderately confident	6 (22.2%)	7 (25.9%)	11 (40.7%)	13 (48.2%)	14 (51.9%)
(4) Very confident [4]	7 (25.9%)	10 (37.0%)	1 (3.7%)	4 (14.8%)	7 (25.9%)
(5) Extremely confident	1 (3.7%)	3 (11.1%)	0 (0%)	1 (3.7%)	2 (7.4%)
Observed score range (min-max)	1–5	1–5	1–4	1–5	1–5
Mean score (SD)	2.7 (1.2)	3.3 (1.1)	2.3 (0.8)	2.7 (1.0)	3.2 (0.9)
Item-scale correlation coefficient	0.58	0.58	0.52	0.20	0.37

### Correlations between measures (Table 5, Fig. 1)

No significant correlations were found between yoga posture self-efficacy scale scores (Y-SEA) and expert-observed yoga competency ratings. Additionally, no significant correlations were found between posture self-efficacy scale scores and practice adherence self-efficacy (Y-PASE) scores.

**Table 5:** Correlations between yoga self-efficacy and competency measures and selected participant characteristics at week 12

	Yoga Posture Self-Efficacy Assessment (Y-SEA) – mean rating for all postures		Expert Observed Competency Assessment – mean rating for all postures		Yoga Practice Adherence Self-Efficacy (Y-PASE)	
	r	*p*-value	r	*p*-vaule	r	*p*-value
Yoga Posture Self-Efficacy Assessment^a^ – mean rating for all postures	1.0		−0.01	0.95	0.22	0.28
Expert Observed Competency Assessment^b^ – mean rating for all postures	−0.01	0.95	1.0		−0.32	0.13
Yoga Practice Adherence Self-Efficacy (Y-PASE)^c^	0.22	0.28	−0.32	0.13	1.0	
Age	−0.63	0.01*	0.19	0.56	−0.38	0.15
Change in urinary incontinence frequency^d^	−0.22	0.79	−0.1	0.66	−0.35	0.08
Patient Perception of Bladder Condition (PPBC)^e^	0.49	0.01*	−0.06	0.80	−0.03	0.87
Hospital Anxiety and Depression Scale-Depression Subscale^f^	−0.34	0.08	0.19	0.39	−0.33	0.09
Hospital Anxiety and Depression Scale-Anxiety Subscale^g^	0.17	0.39	−0.08	0.71	−0.07	0.74
PROMIS Adult Physical Function Profile short-term^h^	−0.41	0.03*	0.18	0.40	−0.17	0.40
Short Physical Performance Battery^i^	0.49	0.01*	−0.06	0.80	−0.03	0.87

*significance at least 0.05

^a^ Participants rated their self-efficacy in performing and holding each of the yoga postures for 30 s on a scale of 1 (not at all confident) to 5 (extremely confident)

^b^ An expert yoga consultant rated each participants’ success in performing each posture on a scale of 1 (not at all successful) to 5 (extremely successful)

^c^ Participants indicated how confident they were that they could practice yoga at home when they: 1) are tired, 2) are in a bad mood, 3) have limited time, 4) are away from home, and 5) are not regularly attending yoga classes

^d^ Absolute change in urinary incontinence frequency between baseline and 12 weeks

^e^ Participants completed the Patient Perception of Bladder Condition (PPBC), a single-item measure assessing the degree to which respondents consider their bladder condition to be a problem

^f^ The Hospital Anxiety and Depression Scale (HADS) – Depression Subscale, a 7-item measure of depression, in which higher scores indicate greater depression symptoms. ^g^ Participants completed the Hospital Anxiety and Depression Scale (HADS) – Anxiety Subscale, a 7-item measure of cognitive anxiety, in which higher scores indicate greater anxiety

^h^ Participants completed the PROMIS Adult Physical Function Profile short-form, a measure for activities of daily living, lower extremity, and central body functions

^I^ Participants completed the Short Performance Physical Battery, a series of physical performance tests to assess lower extremity functioning, with higher scores indicating better functioning

**Fig. 1:**
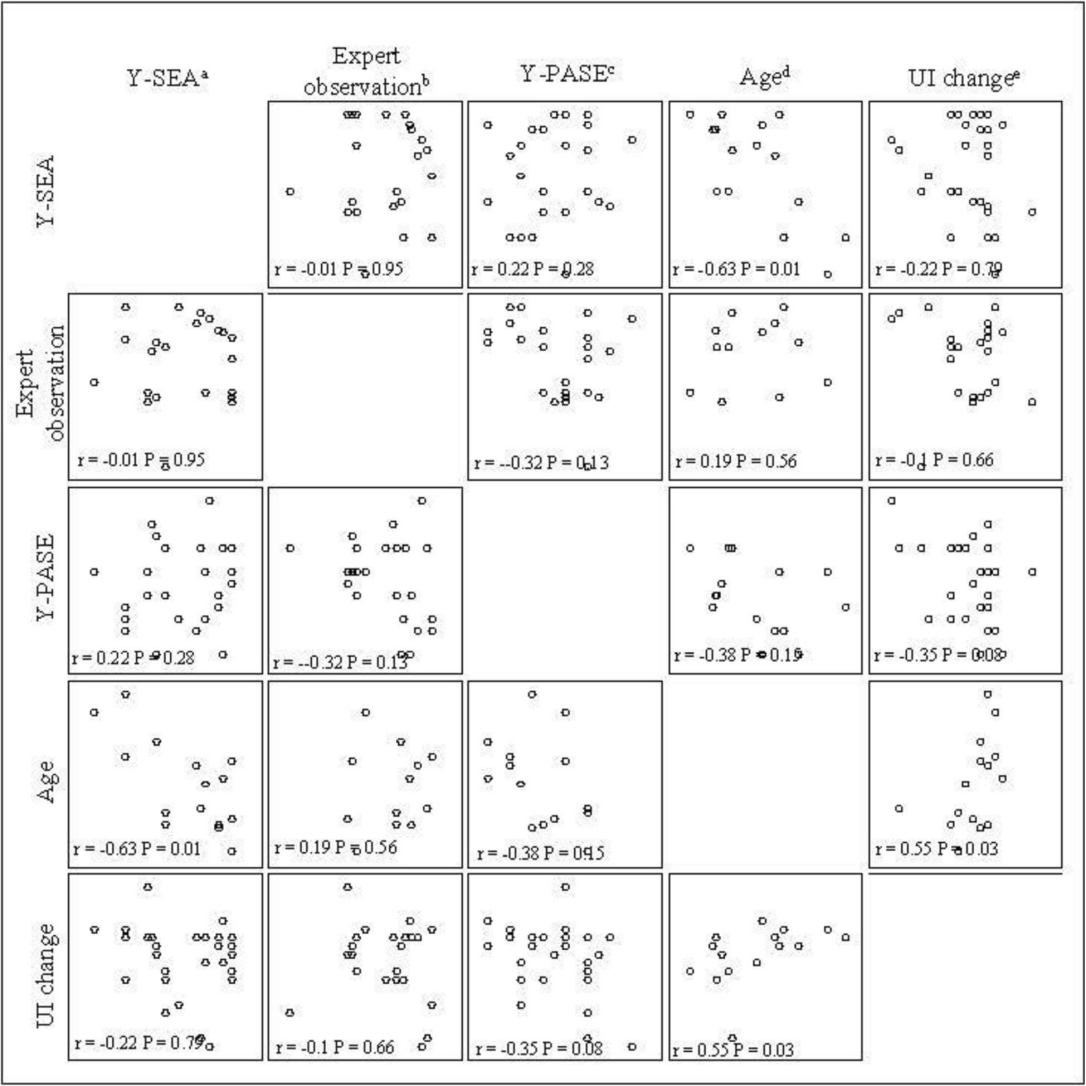
Correlations Between Yoga Self-Efficacy and Competency Measures and Selected Participant Characteristics at Week 12. ^a^ Yoga Posture Self-Efficacy Assessment (Y-SEA) – mean rating for all postures. Participants rated their self-efficacy in performing and holding each of the yoga postures for 30 s on a scale of 1 (not at all confident) to 5 (extremely confident). ^b^ Expert Observed Competency Assessment – mean rating for all postures. An expert yoga consultant rated each participants’ success in performing each posture on a scale of 1 (not at all successful) to 5 (extremely successful). ^c^ Yoga Practice Adherence Self-Efficacy (Y-PASE). Participants indicated how confident they were that they could practice yoga at home when they: 1) are tired, 2) are in a bad mood, 3) have limited time, 4) are away from home, and 5) are not regularly attending yoga classes. ^d^ Participant age at baseline. ^e^ Absolute change in urinary incontinence frequency between baseline and 12 weeks

Yoga posture self-efficacy scores were negatively correlated with participant age (*p* = 0.01), (i.e., older participants demonstrated lower posture self-efficacy) and positively correlated with self-reported physical function assessed by the PROMIS short-form questionnaire (*p* = 0.03) and physical performance/mobility as assessed by the SPPB (p = 0.01), (i.e., participants with higher physical function tended demonstrated higher posture self-efficacy). There was also a non-significant trend toward an inverse correlation between posture self-efficacy score and HADS Depression subscale score (*p* = 0.08), such that those with lower yoga posture self-efficacy scored higher on depression.

No significant correlations were found with any demographic or clinical participant characteristics and expert-observed yoga competency ratings or practice adherence (Y-PASE) ratings. However, there were non-significant trends toward positive correlations between Y-PASE and absolute change in incontinence frequency over 12 weeks (p = 0.08) and depression symptoms at 12 weeks (*p* = 0.09).

## Discussion

Currently, few studies examining yoga as a strategy for treating health conditions describe specific elements of yoga used in their protocols, nor do these studies measure perceived self-efficacy or observed competency regarding the physical performance of specific yoga postures. For this pilot randomized trial of therapeutic Iyengar-based yoga for urinary incontinence in older women, we developed three new tools and present data to evaluate posture and home practice self-efficacy as well as observed competence in the physical performance of yoga postures. Although the pilot trial demonstrated feasibility based on overall retention and adherence, [17] our new analyses of data from the posture self-efficacy and observed competence measures provide insight into unique constructs that may be informative when assessing whether study participants are able to learn to practice physical aspects of yoga and/or maintain this practice over time.

The Yoga Posture Self-Efficacy Assessment (Y-SEA) was designed to measure self-efficacy in performing yoga postures among a population of older women who were relatively naïve to yoga before the start of the study. Responses indicated that only a minority of participants who completed the 12-week yoga program were highly confident in their ability to perform all yoga postures by the end of the program. However, almost three-quarters (74%) indicated that they were at least moderately confident about performing all yoga postures.

Posture self-efficacy ratings varied between postures and category of postures. The three postures with the highest average self-efficacy ratings were Savasana (corpse pose), Tadasana (mountain pose), and Supta padangusthasana (reclined hand to big toe pose), all of which are performed with straight legs. The three most challenging poses according to participants’ self-rating were Malasana (garland/squat pose), Shalabhasana (locust post), and Utkatasana (chair pose). Participants were not asked to indicate the specific aspects of the postures that were difficult or easy. However, common components of these poses that might impact perceived self-efficacy or contribute to feelings of being challenged include a combination of knee, hip, and ankle flexion along with quadricep strength required in Malasana and Utkatasana, which are both standing poses. Shalabhasana, a prone back extension in which the arms, chest, and legs are lifted off the ground, is also a challenging pose in that it requires (but also develops) upper back strength and flexibility and can cause discomfort the lumbar spine if not performed properly.

Although interventions involving physical activity routinely evaluate participants’ success in performing physical aspects of exercise-based protocols, studies focused on therapeutic applications of yoga asana do not often focus on this aspect. In one study, researchers adapted an existing exercise self-efficacy scale for perception of exercise exertion [14]. However, they did not assess self-efficacy or competence regarding physical performance of specific yoga postures. In another study, researchers developed the Yoga Self-Efficacy Scale (YSES) to assess self-efficacy with relation to “body, breath, and mind.” However, the YSES was developed among experienced yoga practitioners and teachers with over 80% white and female, and more than 50% were yoga teachers. The validity of YSES among those with less experience with yoga, including study participants who may be learning yoga technique de novo to assess the therapeutic effects of yoga to treat health conditions, is unknown.

Participants’ observed competence in performing yoga was also moderately to very high, with over one quarter of participants rated by an expert yoga consultant as being very successful in performing all postures. However, the mean expert-observed competency scores had a wider range (3.3–5.0) than participants’ mean posture self-efficacy scores (3.6–4.5), suggesting that the expert observer was both more generous and critical in rating performance of postures than participants. The three postures associated with the highest expert observer competency ratings were the same postures associated with the highest participant posture self-efficacy scores: Savasana (corpse pose), Supta padangusthasana (reclined hand to big toe pose), and Tadasana (mountain pose). However, there were notable differences between the postures associated with the lowest participant self-efficacy ratings and the postures associated with the lowest expert observer competency ratings. According to the expert observer, the least successfully performed postures were Trikonasana (triangle pose), Virabhadrasana 2 (warrior 2), and Salamba setubhandasana (supported bridge pose). Trikonasana and Virabhadrasana 2 are both lateral standing poses that require and develop stability, strength, and flexibility in the lower limbs, including hip flexion and external rotation. Salamba setubhandasana is a supported, supine back extension. Shalabhasana and Salamba setubhandasana, which participants rated as one of the three most challenging poses, are both back extensions which require flexibility in the thoracic spine and shoulder joint. In general, the most challenging categories of postures according to both participants and the expert observer were standing poses and back extensions.

Few studies have used an expert observer to assess the competency of participants in the physical performance of yoga postures. Challenges of this method include the need to arrange for an expert observer to evaluate each participant in person and in real time for each posture included in the study program. Videotaping of group classes has been used as a method to assess fidelity in intervention research [31–33] and may offer an alternative method for assessing participants’ observed success in performing yoga postures. However, potential drawbacks of videotaping include disruption to the yoga classes increased self-consciousness on the part of participants who may object to being recorded. Additionally, an in-person expert observer can move around the room and observe students from multiple angles, thus increasing the potential validity of observed assessment.

Previous research indicates that frequency of home practice maybe a stronger predictor of health outcomes than class frequency [34–36] yet most yoga interventions either do not report home practice or describe measures of home yoga practice adherence [3]. A previous study of a Viniyoga intervention among breast cancer survivors also adapted a self-efficacy scale for exercise to measure self-efficacy for practicing yoga when faced with similar barriers (e.g., when tired, lack of time); in that study population, self-efficacy for practicing yoga was a predictor of adherence to both in-class and home yoga practice [12, 37]. Self-efficacy has also been shown to be a determinant of engaging in physical activity and correlates with adherence to behavioral interventions for incontinence and other health conditions [38–42]. Although the findings from our relatively small pilot study did not provide definitive evidence of a connection between practice adherence self-efficacy and clinical outcomes, future research in larger samples may indicate whether practice adherence self-efficacy is important in determining whether yoga interventions are successful in bringing about the desired health outcome.

Notably, participants’ yoga posture self-efficacy scores were inversely correlated with age, indicating that older study participants had lower self-confidence in performing some physical aspects of the yoga program. Older adults are often underrepresented in clinical trials, particularly trials involving physical-based interventions, including yoga [43–45]. Although the LILA study was successful at recruiting and retaining an older population, [17] these results suggests that older participants in yoga interventions might benefit from more direct encouragement and positive feedback from instructions to improve their confidence in practicing yoga.

Participants’ self-reported physical function tended to have higher positive correlations with posture self-efficacy; however, no similar correlation was found between physical function and the expert observer’s ratings. This suggests that although some participants might feel less confident in their abilities, they were not judged to be less competent by an independent observer. Achieving proper form and function is important when learning and executing new motor skills, particularly for skills intended to improve biomechanical or physiological functioning such as physical yoga postures [46]. When learning new yoga postures for therapeutic purposes, an outside observer might be able to provide a more objective assessment of whether or not the desired form and function was achieved. Those with lower perceived physical function might benefit from encouragement and positive feedback about their ability to perform yoga postures, including instruction in the use of props and modifications, as well as encouragement to accept that these variations are just as valid as classical variations of the postures.

Although we did not find significant correlations between participants’ posture self-efficacy and expert ratings of participants’ competency in performing postures, both are potentially important measures to examine in relation to participant characteristics, intervention effectiveness, and outcomes. When administered early in yoga interventions, self-efficacy process measures may be useful in alerting instructors to provide more support, guidance, and/or encouragement to participants with lower self-efficacy and in turn, promote adherence and retention. Similarly, expert observer ratings of participants’ success in performing postures could be used to help instructors gauge the effectiveness of their instruction or alert them to the need to tailor their approach to instruction. Lack of correlation might also be due to small sample size and low variability within the pilot study. Sample size may also have limited power for factor analysis of the Y-PASE questionnaire, although our sample size is consistent with prior published recommendations that the number of observations be at least five times greater than the number of variables [47]. Assessment of convergent-divergent validity in this modest sample and internal consistency reliability of the posture self-efficacy and observed competency scales (Cronbach’s alpha = 0.93 each) provides preliminary support for their use as multi-item scales. Further evaluation of these measures as well as relationship between these measures and other clinical outcomes should be explored in larger, more diverse samples.

Limitations of this study include its small sample size, which decreased power for determining associations between self-efficacy and home practice adherence measures and clinical outcomes. Yoga posture self-efficacy measure (Y-SEA), expert observer competency assessments, and home practice adherence (Y-PASE) measures were administered at the end of the intervention period (week 12), so we were unable to compare progress from baseline data/assess pre-post change. Reducing participant burden influenced the decision to administer these measures at week 12. In addition, the value of administering self-efficacy measures at the beginning of a study is unclear, since participants may not be able to gauge their ability to perform physical yoga postures or adhere to home practice prior to having received instruction. Similarly, asking participants to perform yoga postures (to assess competency) may not be safe or appropriate before they have received instruction. Future studies might also benefit from qualitative inquiry with study participants to explore individual self-efficacy ratings and explanation of specific aspects of yoga poses that are more or less challenging. In addition, interviews with study participants and instructors could inform best practices and methods to encourage participants with lower posture self-efficacy and/or lower perceived or observed physical function.

While this report focuses on the physical aspects of yoga, we recognize that yoga is a multifaceted practice with interrelated components related to physical, mental and emotional wellbeing. When taught as outlined in classical texts such as the Yoga Sutras of Patanjali, asana (physical postures) is the third of eight limbs of yoga, which also include yama and niyama (ethical precepts toward self and others), pranayama (breath control), pratyahara (withdrawal of the senses), dharana (concentration), dhyana (meditation), and samadhi (meditative absorption) [48]. While beneficial psychological and emotional outcomes may increase by an integrated practice of the eight limbs of yoga, it is also important to understand if and how proper form and function of specific postures and categories of postures (e.g., standing postures, inversions, forward and backward extensions, twisting, and restorative postures) may contribute to the benefits of yoga for particular health conditions. This is especially important for older adults, as prior research suggests that this population, in particular, faces greater challenges in learning the physical rather than the mental aspects of yoga [49, 50]. Without assessments tools such as the ones proposed in this report, we cannot confirm whether success in achieving physical form and function is in fact necessary for reaping the therapeutic benefits of yoga, or explore whether it is more important for some types of health conditions than others.

## Conclusion

This report describes several measures designed to assess self-efficacy and competency in performing yoga postures for therapeutic purposes in the context of a pilot randomized trial of a therapeutic Iyengar-based yoga intervention for urinary incontinence in middle-aged and older women. These measures hold promise for advancing yoga research methods to: 1) measure self-efficacy in physical performance of specific yoga postures; 2) use an expert observer to assess study participants’ competence in performing yoga postures; and 3) measure self-efficacy in adherence to home practice of yoga. These proposed measures can be used early in studies to inform instructors about participants (e.g., those who may need encouragement to fully benefit from yoga interventions) and to assess whether study participants are able to learn to practice physical aspects of yoga and/or maintain this practice over time.

## Supplementary information

**Additional file 1.Table S1**. List of yoga postures featured in the LILA pilot study yoga program.

## Data Availability

The datasets used and/or analysed during the current study are available from the corresponding author on reasonable request.

## Abbreviations

HADS: Hospital Anxiety and Depression Scale
LILA: Lessening Incontinence through Low-Impact Activity study
NCCIH: National Center for Complementary and Integrative Health
PPBC: Patient Perception of Bladder Condition
PROMIS SF: Patient Reported Outcome Measurement Information System Short Form
SPPB: Short Physical Performance Battery
Y-PASE: Yoga Practice Adherence Self-Efficacy
Y-SEA: Yoga Posture Self-Efficacy Assessment
YSES: Yoga Self-Efficacy Scale
