# Supplementary material for: Strategies for evaluating self-efficacy and observed success in the practice of yoga postures for therapeutic indications: methods from a yoga intervention for urinary incontinence among middle-aged and older women
Source: BMC Complement Med Ther. 2020 May 14;20:148. doi: 10.1186/s12906-020-02934-3 (PMC7227071; doi:10.1186/s12906-020-02934-3)
Supplement: Supplementary file 1 — Additional file 1.Table S1. List of yoga postures featured in the LILA pilot study yoga program. [file 12906_2020_2934_MOESM1_ESM.docx]

**Additional File 1. List of yoga postures featured in the LILA pilot study yoga program**

| **Standing postures** |
| --- |
| Tadasana (mountain pose) |
| Utkatasana (chair pose) |
| Trikonasana (triangle pose) |
| Virabhadrasana 2 (warrior 2 pose) |
| Parsvottanasana (intense side stretch pose) |
| **Seated postures** |
| Malasana (garland, or squat pose) |
| Vajrasana (thunderbolt pose) |
| Bharadvajasana (seated twist pose) |
| Baddha Konasana (bound angle pose) |
| **Back extensions** |
| Shalabhasana (locust pose) |
| Salamba Setu Bandhasana (supported bridge pose) |
| **Supine/Restorative postures** |
| Supta Padangusthasana variations (reclined hand to big toe pose) |
| Supta Baddha Konasana (reclined bound angle pose) |
| Viparita Karani Variation (inverted lake pose) |
| Savasana (corpse pose) |
